# Intravascular emboli is an independent risk factor for the prognosis of stage III colorectal cancer patients after radical surgery

**DOI:** 10.18632/oncotarget.11266

**Published:** 2016-08-12

**Authors:** Qian Pei, Hong Zhu, Fengbo Tan, Nanhui Yu, Zhongyi Zhou, Yuan Zhou, Xiangping Song, Yuqiang Li, Yiming Tao, Sai Zhang, Liling Li, Qingling Li, Haiping Pei

**Affiliations:** ^1^ Department of Gastrointestinal Surgery, Xiangya Hospital, Central South University, Changsha, China; ^2^ Department of Oncology, Xiangya Hospital, Central South University, Changsha, China; ^3^ Department of General Surgery, Xiangya Hospital, Central South University, Changsha, China; ^4^ Institute of Medical Sciences, Xiangya Hospital, Central South University, Changsha, China; ^5^ Department of Pathology, Xiangya Hospital, Central South University, Changsha, China

**Keywords:** intravascular emboli, stage III colorectal cancer, prognosis, clinicopathological characteristics, CD133

## Abstract

Lymphovascular emboli is a prognostic factor in stage II CRC, but the significance of intravascular emboli (IVE) in stage III is unclear. Data from consecutive stage III CRC patients receiving radical surgery between January 2009 and November 2014 were retrospectively collected. The expression of CD133 was tested by immumohistochemical (IHC) staining. The potential prognosis risk factors were tested using univariate and multivariate survival analyses. IVE was significantly associated with CD133 expression (*P* < 0.001), gross tumor morphology (*P* = 0.001), histologic type (*p* < 0.001), lymph node status (pN) (*p* < 0.001), sub-class of stage III (*p* = 0.001), and serum CA199 level (*p* = 0.022). IVE, CD133 expression and lymph node status (pN) were independent risk factors for overall survival (OS) (*p* < 0.001, *p* = 0.003, and *p* = 0.008, respectively) and disease-free survival (DFS) (*p* < 0.001, *p* = 0.004, and *p* = 0.007, respectively) in stage III CRC. IVE might be an independent risk factor for the prognosis of stage III CRC patients after radical surgery. IVE might express a cancer stem cell (CSC) phenotype.

## INTRODUCTION

Colorectal cancer (CRC) is the third most common malignant tumor worldwide, and the cancer-related mortality rate is also as high as forth among all tumors [1]. For AJCC stage III [2] CRC, the five-year survival rate is only approximately 60% [3]. Metastasis is the key cause of mortality from cancer [4, 5]. One of the most important steps in metastasis is cancer cells entering the blood circulation and surviving under harsh circumstances [6]. In contrast to single cancer cells, cancer cell clusters, also called cancer emboli, are more effective in overcoming these dangers, including hemodynamic sheer forces, anoikis (a type of apoptosis triggered by loss of anchorage to the substratum) [7, 8] and predation by cells of the innate immune system [6]. Lymphovascular emboli, also known as lymphovascular invasion, is a risk factor for the prognosis of AJCC stage II CRC [9]. However, the significance of lymphovascular emboli, especially intravascular emboli (IVE) in stage III CRC, remains unclear.

Cancer stem cells (CSCs) are a small subpopulation of cancer cells in tumor tissues with characteristics of stem cells, such as the capacity for self-renewal, multi-lineage differentiation and infinite multiplication [10, 11]. According to the CSC hypothesis, CSCs play a pivotal role not only in tumorigenesis but also in almost all of the steps of metastasis [5, 11, 12]. CSCs might present in lymphovascular emboli in certain tumors, such as breast cancer [13, 14].

CSCs have been isolated from a variety of tumors, including CRC [15, 16]. There are many potential CRC stem cell markers, such as CD133, CD44, CD24, CD166, LGR-5 and ALDH-1 [16, 17]. CD133, a five-transmembrane glycoprotein, was originally used to identify CRC stem cells by Lucia Ricci-Vitiani et al [18]. A number of previous studies have demonstrated that CD133 is a reliable CRC stem cell marker [16, 19].

In our study, we investigated the influence of IVE and CSCs on the prognosis of stage III CRC patients who received radical surgery and adjuvant chemotherapy, and we analyzed the relationship between IVE and CSCs. The evaluation of CSCs was performed by CD133 immumohistochemical (IHC) staining.

## RESULTS

### Clinicopathological characteristics associated with intravascular emboli (IVE) status and CD133 expression

In our study, IVE were observed in 131 (40.56%) specimens. Our analysis revealed that IVE were significantly associated with gross tumor morphology (*P* = 0.001), histologic type (*P* < 0.001), lymph node status (pN) (*P* < 0.001), sub-class of stage III (*P* = 0.001), serum CA199 level (*P* = 0.022). There were no associations of IVE with gender, age, tumor site, tumor size, surgery, intestinal obstruction, tumor invasion (pT), serum CEA or CA242 levels (Table 1).

Of the 323 specimens, 144 (44.58%) were CD133 positive (Figure 2). Our analysis revealed that positive expression of CD133 was significantly associated with gross tumor morphology (*P* = 0.034), histologic type (*P* = 0.008), tumor invasion (pT) (*P* = 0.012), lymph node status (pN) (*P* < 0.001), sub-class of stage III (*P* < 0.001), but it was not associated with gender, age, tumor site, tumor size, surgery, intestinal obstruction, serum CEA, CA199 or CA242 levels (Table 1).

**Table 1:** Correlations among intravascular emboli (IVE), CD133 expression and clinicopathological characteristics.

Parameters	IVE		*P* value	CD133		*P* value
	No	Yes		Negative	Positive	
Gender						
Male	103	82	0.110	100	85	0.568
Female	89	49		79	59	
Age(years)						
<65	139	106	0.079	134	111	0.643
≥65	53	25		45	33	
Tumor site^a^						
Right colon	46	25	0.518	43	28	0.429
Left colon	36	29		38	27	
Rectum	110	77		98	89	
Surgery						
Laparoscopic surgery	51	45	0.133	47	49	0.129
Open surgery	141	86		132	95	
Gross tumor morphology						
protruded	133	64	0.001	119	78	0.034
ulcerative	55	58		56	57	
infiltrating	4	9		4	9	
Tumor size (cm)						
<5	125	82	0.644	114	93	0.867
≥5	67	49		65	51	
Intestinal obstruction						
No	178	113	0.057	158	133	0.221
Yes	14	18		21	11	
Histologic type^b^						
Well/moderate	145	65	<0.001	127	83	0.008
Poor	16	51		26	41	
mucinous, SRCC, mix	31	15		26	20	
Tumor invation (pT^c^)						
pT1-pT2	26	10	0.098	27	9	0.012
pT3-pT4	166	121		152	135	
Lymph node status (pN^c^)						
pN1	128	54	<0.001	135	47	<0.001
pN2	64	77		44	97	
Sub-class of stge III^c^						
III A	22	6	0.001	23	5	<0.001
III B	113	62		120	55	
III C	57	63		36	84	
serum cancer markers levels						
CEA (ng/ml)						
<5.00	140	93	0.705	132	101	0.473
≥5.00	52	38		47	43	
CA199 (KU/L)						
<35.00	168	102	0.022	156	114	0.054
≥35.00	24	29		23	30	
CA242 (KU/L)						
<20.00	163	105	0.265	154	114	0.103
≥20.00	29	26		25	30	

a Right colon included the cecum, ascending colon, hepatic flexure, and transverse colon; left colon included the splenic flexure, descending colon, and sigmoid colon.

b Good/moderate, high/moderately differentiated adenocarcinoma; poor, low differentiated adenocarcinoma; SRCC, signet-ring cell carcinoma; mix, adenocarcinoma and mucinous adenocarcinoma or SRCC.

c pT, pN and sub-class of stage III were judged according to the AJCC^7th^ classification.

**Figure 1:**
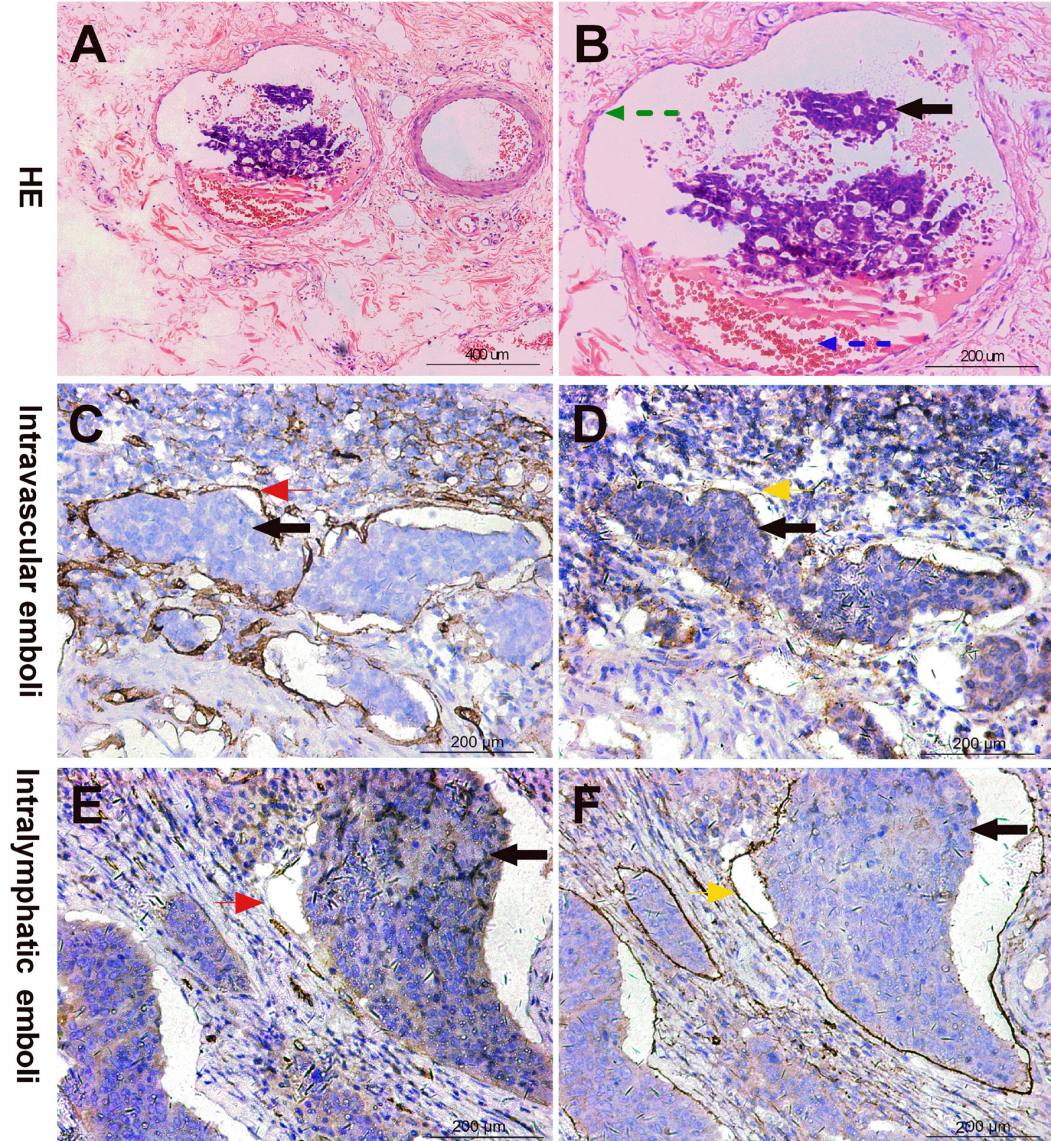
Intravascular emboli (IVE) diagnostic criteria by HE and IHC staining **A**, **B**. IVE (black arrow) diagnosed by HE staining: a cluster of tumor cells in an endothelium-lined space either surrounded by a rim of smooth muscle (green arrow) or containing red blood cells (blue arrow); **C**, **D**. IVE (black arrow) diagnosed by IHC staining: blood vessel endothelium strongly stained by CD34 antibody (red arrow) but not stained by D2-40 (yellow arrow); **E**, **F**. Intralymphatic emboli (black arrow) diagnosed by IHC staining: lymphatic vessel endothelium strongly stained by D2-40 antibody (yellow arrow) but not stained by CD34 (red arrow).

### Correlation between IVE and CD133 expression

In IVE group, 80 (61.07%) were CD133 positive, and 51 (38.93%) were CD133 negative. While, in non-IVE group, 64 (33.33%) were CD133 positive, and 128 (66.67%) were CD133 negative. The χ2 test revealed that IVE was significantly associated with CD133 expression (*P* < 0.001).

### Survival analysis

All the patients were followed up to assess IVE, CD133 expression and clinicopathological characteristics as possible prognostic factors. Of the 323 patients, 109 had metastasis (no-IVE group, 33 *versus* IVE group, 76, *p* < 0.001), and the DFS rate was 66.25%. 84 had cancer-related deaths, and the OS rate was 73.99%. Univariate survival analysis revealed that IVE, CD133 expression, gross tumor morphology, histologic type, pN, sub-class of stage III, and serum CEA, CA199, and CA242 levels were significant prognostic indicators for OS. In multivariate analysis, only IVE (*P* < 0.001), positive expression of CD133 (*P* = 0.003) and pN (*P* = 0.008) remained statistically significant for OS.

Univariate survival analysis revealed that IVE, CD133 expression, gross tumor morphology, histologic type, pN, sub-class of stage III, and serum CA199 and CA242 levels were significant prognostic indicators for DFS. However, in multivariate analysis, only IVE (*P* < 0.001), positive expression of CD133 (*P* = 0.004) and pN (*P* = 0.007) remained statistically significant for DFS (Table 2) (Figure 3).

**Table 2:** Univariate and multivariate analysis of 323 CRC patients for OS and DFS

	OS				DFS			
	Univariate		Multivariate		Univariate		Multivariate	
Variables	HR(95% CI)	*P* Value	HR(95% CI)	*P* Value	HR(95% CI)	*P* Value	HR(95% CI)	*P* Value
Gender (male versus female)	1.353 (0.873-2.097)	0.177	NA		1.262 (0.860-1.854)	0.235	NA	
Age, years (≥65 versus <65)	1.412 (0.883-2.258)	0.150	NA		1.073 (0.698-1.651)	0.747	NA	
Tumor set								
right colon versus left colon	1.229 (0.629-2.402)	0.546	NA		1.017 (0.558-1.853)	0.956	NA	
rectum versus left colon	1.283 (0.719-2.290)	0.399	NA		1.236 (0.749-2.039)	0.407	NA	
rectum versus right colon	1.044 (0.620-1.757)	0.872	NA		1.215 (0.755-1.954)	0.422	NA	
Surgery (laparoscopic versus open)	1.158(0.715-1.876)	0.552	NA		1.156(0.764-1.749)	0.494	NA	
Gross tumor morphology								
ulcerative versus protruded	1.120 (0.682-1.839)	0.653	NA		1.431 (0.954-2.147)	0.083	NA	
infiltrating versus protruded	3.175 (1.500-6.721)	0.003	1.444 (0.654-3.188)	NS	3.448 (1.705-6.975)	0.001	1.456 (0.693-3.060)	NS
infiltrating versus ulcerative	2.834 (1.264-6.353)	0.011	2.032 (0.887-4.652)	NS	2.409 (1.166-4.979)	0.018	1.557 (0.740-3.274)	NS
Tumor size, cm (≥5 versus <5)	1.397 (0.909-2.149)	0.127	NA		1.251 (0.855-1.831)	0.249	NA	
Obstruction (yes versus no)	1.461 (0.775-2.756)	0.241	NA		1.394 (0.781-2.489)	0.261	NA	
Histologic type								
mucinous/SRCC/mix versus well/maderate	1.707 (0.918-3.175)	0.091	NA		1.357 (0.781-2.357)	0.279	NA	
poor versus well/moderate	3.119 (1.921-5.065)	<0.001	1.604 (0.928-2.771)	NS	2.436 (1.588-3.737)	<0.001	1.028 (0.641-1.647)	NS
poor versus mucinous/SRCC/mix	1.827 (0.942-3.545)	0.075	NA		1.796 (0.987-3.266)	0.055	NA	
Tumor invation, pT (pT3-4 versus pT1-2)	2.115 (0.856-5.224)	0.104	NA		2.027 (0.943-4.361)	0.071	NA	
Lymph node status, pN (pN2 versus pN1)	3.606 (2.284-5.694)	<0.001	2.151 (1.219-3.797)	0.008	2.810 (1.903-4.150)	<0.001	1.956 (1.201-3.185)	0.007
Sub-class of stage III								
III B versus III A	1.781 (0.546-5.809)	0.338	NA		2.071 (0.747-5.740)	0.162	NA	
III C versus III A	5.222 (1.624-16.788)	0.006	1.239 (0.291-5.267)	NS	4.382 (1.588-12.094)	0.004	1.264 (0.372-4.293)	NS
III C versus III B	2.931 (1.876-4.580)	<0.001	1.205 (0.572-2.536)	NS	2.116 (1.439-3.110)	<0.001	0.868 (0.465-1.619)	NS
serum tumor markers levels								
CEA, ng/ml (≥5.00 versus <5.00)	1.683 (1.083-2.615)	0.021	1.394 (0.843-2.305)	NS	1.302 (0.871-1.946)	0.199	NA	
CA199, KU/L (≥35.00 versus <35.00)	1.917 (1.185-3.101)	0.008	1.000 (0.342-2.925)	NS	1.973 (1.276-3.050)	0.002	1.463 (0.612-3.500)	NS
CA242, KU/L (≥20.00 versus <20.00)	1.769 (1.093-2.862)	0.020	1.378 (0.485-3.916)	NS	1.677 (1.079-2.608)	0.022	1.119 (0.465-2.695)	NS
IVE (yes versus no)	5.758 (3.571-9.285)	<0.001	4.791 (2.916-7.871)	<0.001	5.687 (3.750-8.623)	<0.001	4.806 (3.096-7.462)	<0.001
CD133 expression (positive versus negative)	3.292 (2.088-5.189)	<0.001	2.079 (1.274-3.393)	0.003	2.924 (1.972-4.334)	<0.001	1.863 (1.223-2.839)	0.004

Abbreviations: HR, hazard ratio; CI, confidence interval; NA, not available; NS, not significant.

**Figure 2:**
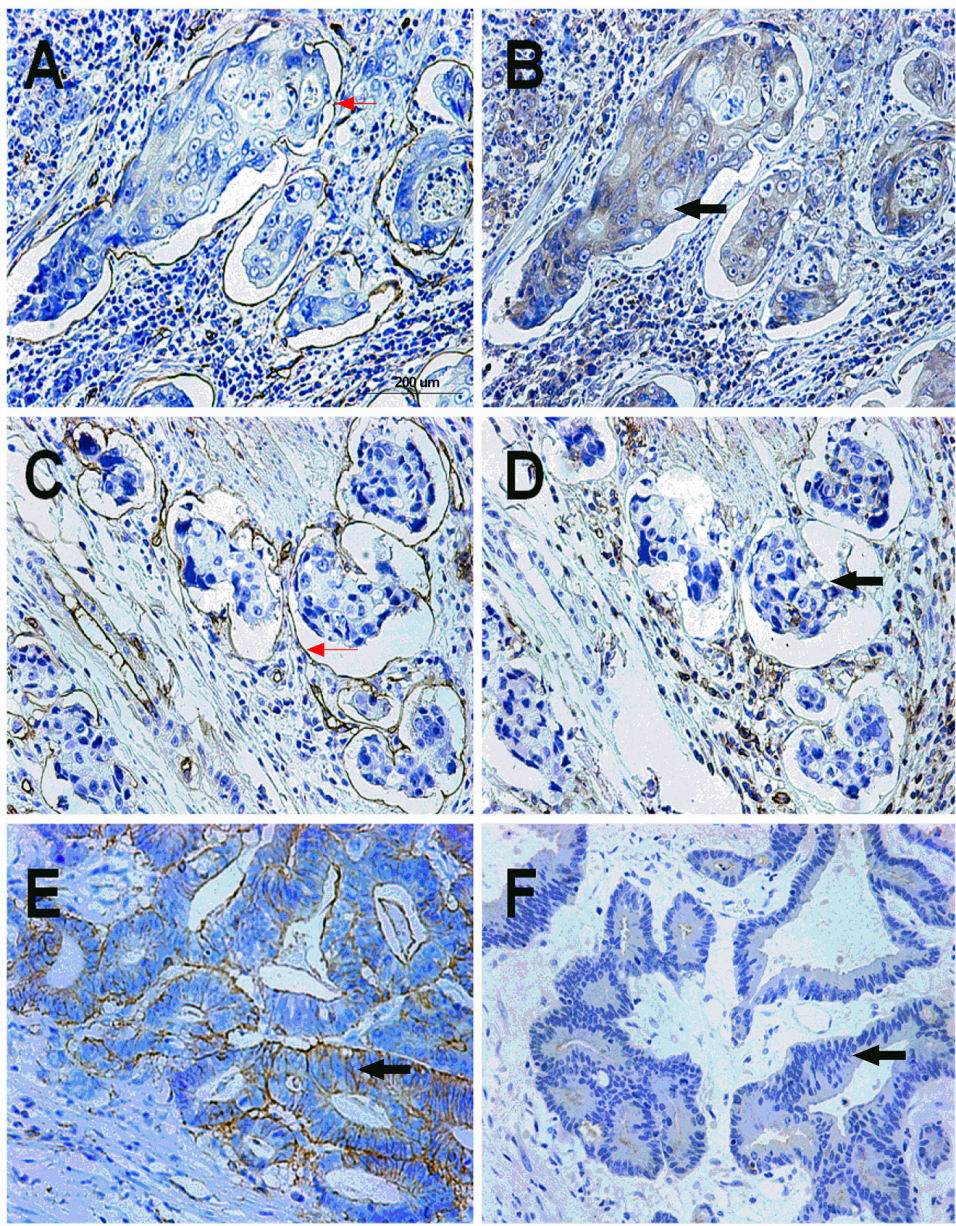
CD133 expression status in IVE and non-IVE colorectal cancer tissues **A**, **B**. Positive expression of CD133 in IVE colorectal cancer tissue: CD34 IHC staining marked the blood vessel endothelium (red arrow), and IVE was strongly stained by CD133 antibody (black arrow); **C**, **D**. CD133-negative expression in IVE colorectal cancer tissue: CD34 IHC staining marked the blood vessel endothelium (red arrow); IVE was not stained by the CD133 antibody (black arrow); **E**. Positive expression of CD133 in non-IVE colorectal cancer tissue (black arrow); F. CD133-negative expression in non-IVE colorectal cancer tissue (black arrow).

**Figure 3:**
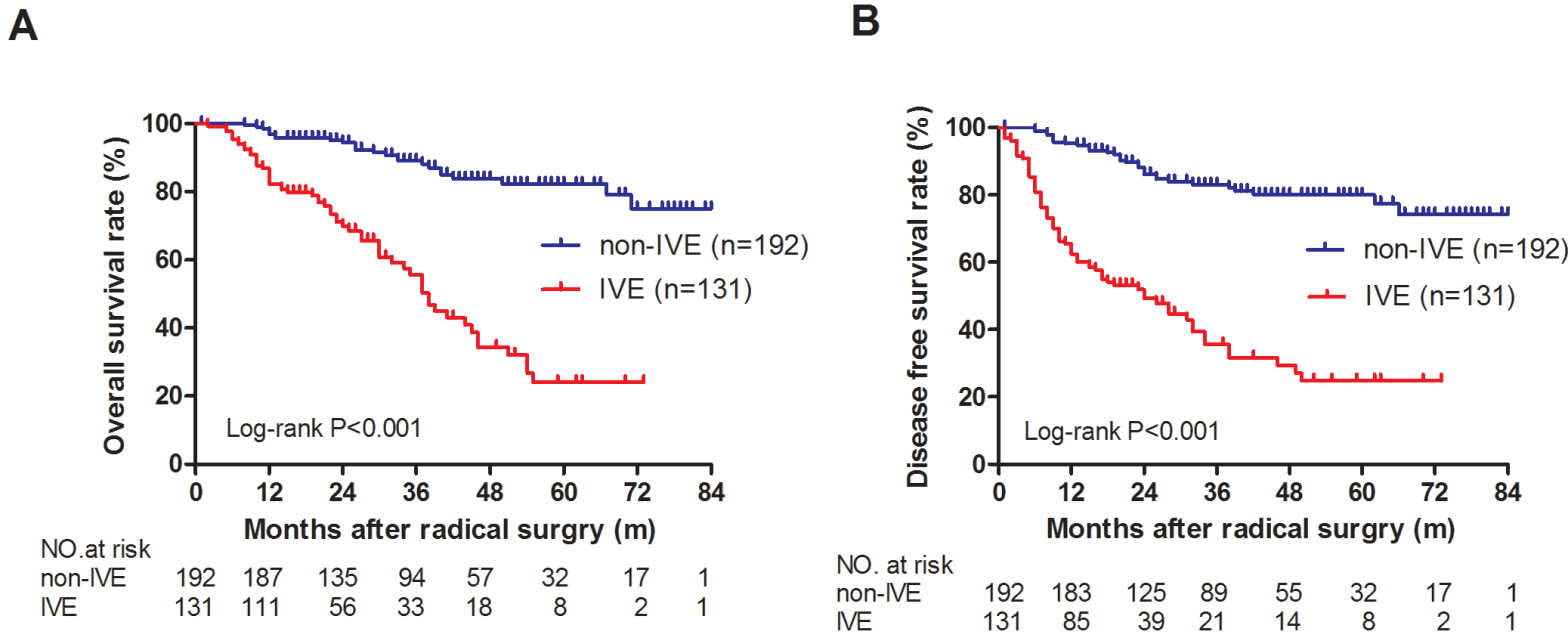
OS and DFS in IVE and non-IVE colorectal cancer patients **A**. OS was significantly longer in non-IVE patients than in IVE patients; **B**. DFS was significantly longer in non-IVE patients than in IVE patients.

## DISCUSSION

Despite receiving radical surgery and adjuvant chemotherapy, the survival rate of stage III CRC patients remains unsatisfactory [3, 20]. Like other solid tumors, metastasis accounts for most deaths [4, 5]. In our study, 84 (26.01%) died from metastasis during follow-up. Therefore, it is necessary to identify the risk factors for poor prognosis in stage III CRC.

For most solid tumors, metastases are formed following a complex succession of cell-biological events: regional invasion entering transport passageways, such as lymphatic and blood vessels; survival in the circulation; arrest at distant organs; extravasation; micrometastasis formation; and metastatic colonization [6, 21]. Several studies have reported that blood circulation, rather than lymph draining, is the main channel for tumor cell dissemination [5, 22]. Cancer cells face a variety of stresses in the circulation. However, by forming relatively larger cancer cell clusters (cancer emboli), they can simultaneously evade these threats efficiently [6, 23]. Thus, we sought to explore the significance of IVE in stage III CRC. Our study results suggested that IVE might be a reliable predictor of survival in stage III CRC patients after radical surgery.

CRC staging systems remain based on the TNM (Tumor Node Metastasis) classification [2]. The TNM classification emphasizes lymphatic invasion, that is, regional invasion. However, the importance of vascular invasion, a potential metastasis predictor, remains obscure. This might explain why these CRC staging systems can’t predict prognosis very satisfactorily [3]. In our study, multivariate survival analysis revealed that the sub-class of stage III based on TNM classification was not an independent prognosis predictor, and the hazard rate of lymph invasion was less than that of vascular invasion. Therefore, we strongly recommend that the vascular invasion, also known as IVE, should be paid sufficient attention.

Cancer cells adapting to foreign environments and proliferating to colonize metastatic sites is the final step in metastasis [21]. This biological behavior coincides with the central trait of CSCs - the ability to seed new tumors when experimentally planted into appropriate animal hosts [5]. In theory, tumor initiation by disseminated cancer cells at metastatic sites is similar to that by experimentally planted cells. Both processes depend on the ability of cancer cells to act as founder cells and to produce unlimited numbers of descendants. CD133 was successfully used to identify sub-populations of CRC cells with the capacity to initiate tumor growth in immunodeficient mice [18, 24].

As a continuous process, metastasis will not succeed if any step is disrupted. In breast cancer, several studies have found that lymphovascular emboli manifest a stem cell phenotype [13, 14]. Therefore, we presumed that the primary tumors and corresponding IVE that successfully metastasize would possess CSCs and would be positive for CD133 expression. In our study, multivariate survival analysis revealed that CD133 expression was also an independent risk factor for DFS and OS in stage III CRC patients. This result was consistent with those of several previous studies [19, 25, 26]. The χ2 test revealed that CD133 expression was significantly associated with IVE. These results supported our hypothesis.

Chemoradiotherapy resistance is another characteristic of CSCs. In our study, all patients received adjuvant chemotherapy after radical surgery, but there were still a proportion of patients developing metastasis. The CSC theory explains this very well [27, 28]. Several previous studies also found that positive expression of CD133 was associated with chemotherapy resistance in CRC [29, 30].

Our study was one of the few studies to assess the influence of IVE on the prognosis of stage III CRC. In our study, IVE was an independent risk factor for the prognosis of stage III CRC patients after radical surgery. IVE was significantly associated with CD133 expression. Greater attention should be paid to IVE in stage III CRC.

## MATERIALS AND METHODS

### Patients and clinical follow-up

We collected the consecutive clinicopathological data of stage III CRC patients in the Gastrointestinal Surgery Department, Xiangya Hospital, Central South University, China, between January 2009 and November 2014. The total number of patients who matched the enrollment criteria was 323 (131 cases in the IVE group and 192 cases in the no-IVE group). All of them received radical surgery (total mesorectal excision, TME for rectal cancer and complete mesocolic excision, CME for colon cancer) and adjuvant chemotherapy (FOLFOX4, 48; CapeOX, 29; FOLFIRI, 10; mFOLFOX6, 236). The enrollment exclusion criteria included: neoadjuvant chemotherapy or neoadjuvant chemoradiotherapy before surgery; colorectal cancer with intestinal perforation; refusal to accept adjuvant chemotherapy based on 5-fluorouracil after surgery; synchronous multifocal colorectal cancer; surgery not reaching R0 excision; and incomplete clinicopathological data. The data on serum tumor markers came from the peripheral venous blood of patients admitted before surgery under fasting conditions. We conducted telephone follow-ups every three months. The deadline of follow-up was February 2016. Disease-free survival (DFS) was defined as the interval from radical surgery to recurrence or metastasis, the appearance of secondary colon or rectal cancer, or death, whichever occurred first. Overall survival (OS) was defined as the interval from radical surgery to mortality, or it was censored at the last known date alive.

### Intravascular Emboli (blood vessel invasion, BVI) diagnostic criteria

An intravascular embolus is “a rounded mass of tumor in an endothelium-lined space either surrounded by a rim of smooth muscle or containing red blood cells” [31]. In our study, a minimum of 4 paraffin tumor blocks were used to optimize detection. When we encountered difficulties in identification and distinguishing sample features from lymphatic vessel invasion (LVI) in hematoxylin and eosin (H &E) slices, we marked the blood vessel endothelium and lymphatic vessel endothelium, with CD34 and D2-40 IHC staining, respectively, in serial sections [32, 33]. Because several researchers have reported that a few of the lymphatic vessel endothelium also could be weakly stained by CD34 antibody, we defined the vessel endothelium as the blood vessel endothelium when the staining of CD34 was moderate or strong [34]. All of the slices were simultaneously assessed by two independent pathologists, who then compared their results and jointly reviewed controversial slices. Only the cases on which both pathologists agreed were judged as positive for IVE and were recorded on the pathology report (Figure 1).

### Immumohistochemical staining

Formalin-fixed, paraffin-embedded archived colorectal cancer surgical specimens were sectioned at a thickness of 4 μm in the Pathology Department, Xiangya Hospital, Central South University, Changsha, China. The steps are listed briefly as follows. First, the sections were dewaxed and rehydrated. Then, the sections were immersed in 10 mmol/l sodium citrate buffer (pH = 6), incubated for 10 min on a hot plate (95-99°C) or boiled and allowed to cool for 20 min to repair the sealed antigen. The sections were then incubated in 3% hydrogen peroxide solution for 25 minutes to block endogenous catalase. After blocking with 3% BSA, the sections were incubated with rabbit polyclonal antibody against human CD133 at a dilution of 1:200 (18470-1-AP, Proteintech, China) overnight at 4°C, and this was followed by incubation with secondary antibody for 50 minutes at room temperature. The sections were stained with 0.05% diaminobenzidine tetrahydrochloride (DAB) for 5 minutes and then counterstained with hematoxylin, dehydrated and mounted. Negative control sections were stained by omitting the primary antibody.

The specimens were evaluated by two observers who were blinded to the prognosis or other clinicopathological variables. We used a semi-quantitation system for the intensity of staining and the percentage of positive cells. The samples were grouped into the following four categories, based on the intensity of membrane staining: 0. no staining/background equal to the negative controls; 1. weak staining (faint yellow) detectable above the background; 2. moderate staining (yellow); and 3. intense staining (tawny). The labeling frequency was scored as 0 (0%), 1 (1-33%), 2 (34%-66%) or 3 (67-100%). The index sum was obtained by totaling the score for intensity and the percentage. If the final score was equal to or greater than 4, the result was considered positive; otherwise, the result was considered negative. Each slice was randomly verified under 5 high power fields (HPF, ×400), and the average was calculated for scoring.

### Statistical analysis

The associations among IVE status, CD133 expression and clinicopathological parameters were evaluated using the Chi-square (χ2) test. When the subset sample size was small, the corresponding test was conducted using Fisher's exact test. DFS and OS curves were plotted according to the Kaplan-Meier method; A Cox regression model was used to perform multivariate analysis, parameters of significance in univariate analysis were included in the multivariate analysis. All of the analyses were conducted using SPSS 13.0 software (SPSS, Inc., Chicago, IL). *P* < 0.05 was considered to be statistically significant. All of the reported *P*-values were two-sided.

## References

[1] Torre LA, Bray F, Siegel RL, Ferlay J, Lortet-Tieulent J, Jemal A (2015). Global cancer statistics, 2012. CA Cancer J Clin.

[2] Edge SB, Compton CC (2010). The American Joint Committee on Cancer: the 7th edition of the AJCC cancer staging manual and the future of TNM. Ann Surg Oncol.

[3] Hari DM, Leung AM, Lee J, Sim M, Vuong B, Chiu CG, Bilchik AJ (2013). AJCC Cancer Staging Manual 7th Edition Criteria for Colon Cancer: Do the Complex Modifications Improve Prognostic Assessment?. J Am Coll Surgeons.

[4] Chambers AF, Groom AC, MacDonald IC (2002). Dissemination and growth of cancer cells in metastatic sites. Nat Rev Cancer.

[5] Chaffer CL, Weinberg RA (2011). A perspective on cancer cell metastasis. Science.

[6] Valastyan S, Weinberg RA (2011). Tumor metastasis: molecular insights and evolving paradigms. Cell.

[7] Frisch SM, Francis H (1994). Disruption of epithelial cell-matrix interactions induces apoptosis. J Cell Biol.

[8] Paoli P, Giannoni E, Chiarugi P (2013). Anoikis molecular pathways and its role in cancer progression. Biochimica et Biophysica Acta.

[9] Benson AR, Schrag D, Somerfield MR, Cohen AM, Figueredo AT, Flynn PJ, Krzyzanowska MK, Maroun J, McAllister P, Van Cutsem E, Brouwers M, Charette M, Haller DG (2004). American Society of Clinical Oncology recommendations on adjuvant chemotherapy for stage II colon cancer. J Clin Oncol.

[10] Nguyen LV, Vanner R, Dirks P, Eaves CJ (2012). Cancer stem cells: an evolving concept. Nat Rev Cancer.

[11] Beck B, Blanpain C (2013). Unravelling cancer stem cell potential. Nat Rev Cancer.

[12] Dalerba P, Clarke MF (2007). Cancer stem cells and tumor metastasis: first steps into uncharted territory. Cell Stem Cell.

[13] Xiao Y, Ye Y, Yearsley K, Jones S, Barsky SH (2008). The Lymphovascular Embolus of Inflammatory Breast Cancer Expresses a Stem Cell-Like Phenotype. The American Journal of Pathology.

[14] Van Laere S, Limame R, Van Marck EA, Vermeulen PB, Dirix LY (2010). Is there a role for mammary stem cells in inflammatory breast carcinoma?. Cancer-Am Cancer Soc.

[15] Akbari-Birgani S, Paranjothy T, Zuse A, Janikowski T, Cieślar-Pobuda A, Likus W, Urasińska E, Schweizer F, Ghavami S, Klonisch T, Łos MJ (2016). Cancer stem cells, cancer-initiating cells and methods for their detection. Drug Discov Today.

[16] Todaro M, Francipane MG, Medema JP, Stassi G (2010). Colon Cancer Stem Cells: Promise of Targeted Therapy. Gastroenterology.

[17] Gerger A, Zhang W, Yang D, Bohanes P, Ning Y, Winder T, LaBonte MJ, Wilson PM, Benhaim L, Paez D, El-Khoueiry R, El-Khoueiry A, Kahn M (2011). Common Cancer Stem Cell Gene Variants Predict Colon Cancer Recurrence. Clin Cancer RES.

[18] Ricci-Vitiani L, Lombardi DG, Pilozzi E, Biffoni M, Todaro M, Peschle C, De Maria R (2006). Identification and expansion of human colon-cancer-initiating cells. Nature.

[19] Zhao Y, Peng J, Zhang E, Jiang N, Li J, Zhang Q, Zhang X, Niu Y (2016). CD133 expression may be useful as a prognostic indicator in colorectal cancer, a tool for optimizing therapy and supportive evidence for the cancer stem cell hypothesis: a meta-analysis. Oncotarget.

[20] Siegel RL, Miller KD, Jemal A (2016). Cancer statistics, 2016. CA Cancer J Clin.

[21] Nguyen DX, Bos PD, Massagué J (2009). Metastasis: from dissemination to organ-specific colonization. Nat Rev Cancer.

[22] Joyce JA, Pollard JW (2008). Microenvironmental regulation of metastasis. Nat Rev Cancer.

[23] Quail DF, Joyce JA (2013). Microenvironmental regulation of tumor progression and metastasis. Nat Med.

[24] O’Brien CA, Pollett A, Gallinger S, Dick JE (2006). A human colon cancer cell capable of initiating tumour growth in immunodeficient mice. Nature.

[25] Kojima M, Ishii G, Atsumi N, Fujii S, Saito N, Ochiai A (2008). Immunohistochemical detection of CD133 expression in colorectal cancer: a clinicopathological study. Cancer Sci.

[26] Lin EH, Hassan M, Li Y, Zhao H, Nooka A, Sorenson E, Xie K, Champlin R, Wu X, Li D (2007). Elevated circulating endothelial progenitor marker CD133 messenger RNA levels predict colon cancer recurrence. Cancer-Am Cancer Soc.

[27] Greaves M, Maley CC (2012). Clonal evolution in cancer. Nature.

[28] Dallas NA, Xia L, Fan F, Gray MJ, Gaur P, van Buren G, Samuel S, Kim MP, Lim SJ, Ellis LM (2009). Chemoresistant Colorectal Cancer Cells, the Cancer Stem Cell Phenotype, and Increased Sensitivity to Insulin-like Growth Factor-I Receptor Inhibition. Ccancer Res.

[29] Shikina A, Shinto E, Hashiguchi Y, Ueno H, Naito Y, Okamoto K, Kubo T, Fukazawa S, Yamamoto J, Hase K (2014). Differential Clinical Benefits of 5-Fluorouracil-based Adjuvant Chemotherapy for Patients with Stage III Colorectal Cancer According to CD133 Expression Status. JPN J Clin Oncol.

[30] Ong CW, Kim LG, Hui HK, Lai YL, Iacopetta B (2010). CD133 expression predicts for non-response to chemotherapy in colorectal cancer. Modern Pathology.

[31] Talbot IC, Ritchie S, Leighton MH, Hughes AO, Bussey H (1980). The clinical significance of invasion of veins by rectal cancer. Brit J Surg.

[32] van Wyk HC, Roxburgh CS, Horgan PG, Foulis AF, McMillan DC (2014). The detection and role of lymphatic and blood vessel invasion in predicting survival in patients with node negative operable primary colorectal cancer. Critical Reviews in Oncology/Hematology.

[33] Messenger DE, Driman DK, Kirsch R (2012). Developments in the assessment of venous invasion in colorectal cancer: implications for future practice and patient outcome. Hum Pathol.

[34] Liang P, Nakada I, Hong J, Tabuchi T, Motohashi G, Takemura A, Nakachi T, Kasuga T, Tabuchi T (2007). Prognostic Significance of Immunohistochemically Detected Blood and Lymphatic Vessel Invasion in Colorectal Carcinoma: Its Impact on Prognosis. Ann Surg Oncol.

